# Early Determination of the Periodontal Domain by the Wnt-Antagonist Frzb/Sfrp3

**DOI:** 10.3389/fphys.2017.00936

**Published:** 2017-11-21

**Authors:** Thimios A. Mitsiadis, Pierfrancesco Pagella, Claudio Cantù

**Affiliations:** ^1^Orofacial Development and Regeneration, Institute of Oral Biology, Centre for Dental Medicine, Medical Faculty, University of Zurich, Zurich, Switzerland; ^2^Institute of Molecular Life Sciences, University of Zurich, Zurich, Switzerland

**Keywords:** Frzb/Srfp3, Wnt signaling, tooth development, cell fate, dental pulp, dental follicle, periodontium

## Abstract

Odontogenesis results from the continuous and reciprocal interaction between cells of the oral epithelium and cranial neural crest-derived mesenchyme. The canonical Wnt signaling pathway plays a fundamental role in mediating these interactions from the earliest stages of tooth development. Here we analyze by *in situ* hybridization the expression patterns of the extracellular Wnt antagonist *Frzb/Sfrp3*. Although *Frzb* is expressed in dental mesenchymal cells from the earliest stages of odontogenesis, its expression is absent from a tiny population of mesenchymal cells immediately adjacent to the invaginating dental epithelium. Cell proliferation studies using BrdU showed that the *Frzb* expressing and *Frzb* non-expressing cell populations display different proliferative behavior during the initial stages of odontogenesis. DiI-mediated cell-fate tracing studies demonstrated that the *Frzb* expressing cells contribute to the formation of the dental follicle, the future periodontium. In contrast, the *Frzb* non-expressing cells give rise to the dental pulp. The present results indicate that Frzb is discriminating the presumptive periodontal territory from the rest of the dental mesenchyme from the very beginning of odontogenesis, where it might act as a barrier for the diffusion of Wnt molecules, thus regulating the activation of Wnt-dependent transcription within dental tissues.

## Introduction

Odontogenesis is characterized by the sequential and reciprocal interactions between cells of the oral epithelium and the cranial neural crest-derived mesenchyme and proceeds through a series of well-defined morphological steps, namely bud, cap, bell, and cytodifferentiation/mineralization stages (Mitsiadis and Graf, 2009). Ectomesenchymal cells form two distinct and specialized tooth components, the dental papilla that gives rise to the pulp and the dentin-producing odontoblasts, and the dental follicle, which surrounds the developing dental epithelium and forms the periodontium (Mitsiadis and Graf, 2009; Krivanek et al., 2017).

All stages of tooth development are mediated by the exchange of a big variety of signaling molecules between homotypic and heterotypic cell populations (Mitsiadis and Graf, 2009; Mitsiadis and Luder, 2011; Jussila and Thesleff, 2012; Balic and Thesleff, 2015). Among these molecules, the secreted lipid-modified Wnt glycoproteins trigger the evolutionarily conserved Wnt signaling pathway, a molecular cascade important for the development of virtually all organs (Clevers, 2006). One previous study has shown that several extracellular Wnt ligands and Wnt inhibitors are expressed in specific stages and compartments during odontogenesis (Sarkar and Sharpe, 1999). Although many reports addressed the role of the canonical Wnt/β-catenin-mediated signaling in the formation and regeneration of dental tissues (Aurrekoetxea et al., 2012, 2016; Liu et al., 2014; Zhang et al., 2016; Babb et al., 2017) a unifying picture of its activity during odontogenesis is still missing (Tamura and Nemoto, 2016). The critical requirement of Wnt signaling during tooth development has been already evidenced, since the genetic loss of β-catenin, or the specific abrogation of β-catenin-dependent transcription, leads to arrested tooth formation at early stages (Liu et al., 2008; Cantù et al., 2017).

The action of the Wnt ligands in the extracellular matrix is regulated by physiologically secreted Wnt antagonists (or negative regulators) such as the ones belonging to the family of Secreted Frizzled Related Proteins (SFRPs), which possess a cysteine-rich domain homologous to the Wnt-binding domain of Frizzled (Frzb) receptors (Cruciat and Niehrs, 2013) and are implicated as tumor suppressors in several forms of cancer (Zimmerli et al., 2017).

Frzb (known as Frzb1 or Sfrp3, Secreted Frizzled Related Protein 3) was initially identified as a chondrogenic factor during bone morphogenesis (Hoang et al., 1996). It was subsequently shown to modulate the activity of XWnt8 during *Xenopus* dorsoventral axis development (Leyns et al., 1997) and to repress canonical Wnt signaling in other contexts (Person et al., 2005). Here we identify *Frzb* as a novel marker of the neural crest-derived mesenchymal cells that contribute to dental follicle formation, the future periodontium. *Frzb* expression at the earliest stages of odontogenesis allows distinguishing two dental mesenchymal cell populations with clearly defined developmental fates.

## Materials and methods

### Cell proliferation analysis

All animals were maintained and handled according to the Swiss Animal Welfare Law and in compliance with the regulations of the Cantonal Veterinary Office, Zurich (License 11/2014). *In vivo* cell proliferation in dental tissues was analyzed by immunohistochemistry for phosphorylated Histone 3 (pH3; rabbit Ab, 1:200; Upstate, Charlottesville, VA) and bromodeoxyuridine (BrdU). For the latter, a BrdU cell proliferation kit (Boehringer Mannheim, Germany) was used. Foster mothers were injected intraperitoneally with 5 mg/ml of BrdU in PBS at a concentration of 50 mg/kg body-weight, 60 min before embryos were sacrificed. BrdU-positive cells in developing teeth of E13–E15 embryos were analyzed on 14 μm cryosections after staining with an anti-BrdU antibody. Immunohistochemistry was performed as described earlier (Mitsiadis et al., 2008). Cells were counted with the CellCounter Plugin, ImageJ. Statistical Analysis was performed with GraphPad Prism 7 (*t*-test).

### Lineage tracing using dii labeling

DiI (1, 10, di-octadecyl-3, 3, 30,-tetramethylindo-carbocyanine perchlorate; Molecular probes cell tracker CM-DiI, C-7000) injection was performed to various locations of mesenchymal cells surrounding the dental epithelial ingrowths of cultured mandible slices to monitor cell kinetics. Briefly, E13 mouse mandibles were carefully dissected out from the head, placed upon a chopping plate of a McIlwain tissue chopper (Mickle Laboratory Engineering Co., Ltd., Guilford, UK), orientated to obtain frontal sections and finally cut into 250 μm thick slices. Slices containing the molar tooth buds were selected and cultured. DiI, which is highly lipophilic dye intercalating into the cell membranes, was dissolved in ethanol (EtOH) at 2.5 μg/μl (stock solution) and then diluted 1–9 in 0.2 M sucrose. Thereafter, small amounts of DiI were injected using a mouth-controlled micropipette made from a 50 mm borosilicate glass into different areas of the condensing dental mesenchyme, either in mesenchymal cells contacting the molar bud epithelium or in condensing cells located at a more distant area from the tooth epithelium (Mitsiadis et al., 2008; Gruenbaum-Cohen et al., 2009).

### Slice cultures and imaging

DiI labeled slices were placed upon Millipore filters coated with growth factor reduced Matrigel basement membrane matrix (BD Biosciences). The slices were completely encapsulated by an additional layer of Matrigel that served to structurally support the morphology of the explants during their development. The filters were supported above the culture medium by metal grids within Petri dishes. The culture medium was composed of Dulbecco's Minimum Essential Medium (DMEM) supplemented with 1% penicillin/streptomycin, 2 mM L-glutamine and 15% fetal calf serum (FCS). Slices were cultured up to 4 days in a 37°C/5% CO2 air-jacketed incubator. After culturing, samples were fixed in 4% paraformaldehyde (PFA) for 30 min, washed with PBS and then embedded in wax and sectioned at 8 μm.

The initial positions of the DiI injection and the subsequent location of the DiI-labeled cells were monitored throughout the culture period using a Leica dissecting microscope equipped with UV light (Leica Microsystems Ltd., Germany).

### *In Situ* hybridization

*Frzb in situ* hybridization probe was kindly provided by Prof. De Robertis (Leyns et al., 1997). The labeled probe was ethanol-precipitated, resuspended in 100 mM DTT, diluted in hybridization solution (60% deionized formamide, 20 mM Tris-HCl, 5 mM EDTA, pH 8, 0.3 M NaCl, 0.5 mg/ml yeast RNA, 5% dextran sulfate). *In situ* hybridization was performed according to standard procedures (Mitsiadis et al., 1995). Briefly, slides were incubated with the probe at 60°C. After intense washing, the slides were incubated in blocking solution (20% Normal Goat Serum) and anti-digoxigenin (DIG)-AP (alkaline phosphatase conjugate) Fab-fragment (Boehringer Mannheim, 1093 274) diluted 1:1,000 in blocking solution. The color reaction was developed using Nitro Blue Tetrazolium (NBT, Sigma N-6876) and 5-Bromo-4-Chloro-3-Indolyl Phosphate (BCIP, Sigma B-8503) in staining solution 2% NaCl, 5% MgCl2, 10% Tris-HCl pH 9.5, 1% Tween-20. *In situ* hybridization immediately followed by BrdU immunohistochemistry was performed in cryosectioned slides of E13–E15 mouse embryos to show the correlation between *Frzb* expression and cell proliferation (Mitsiadis et al., 2008). No hybridization signal was detected with the sense probe at these developmental stages.

## Results

### *Frzb* is expressed in a subpopulation of dental mesenchymal cells

**Figure 1:**
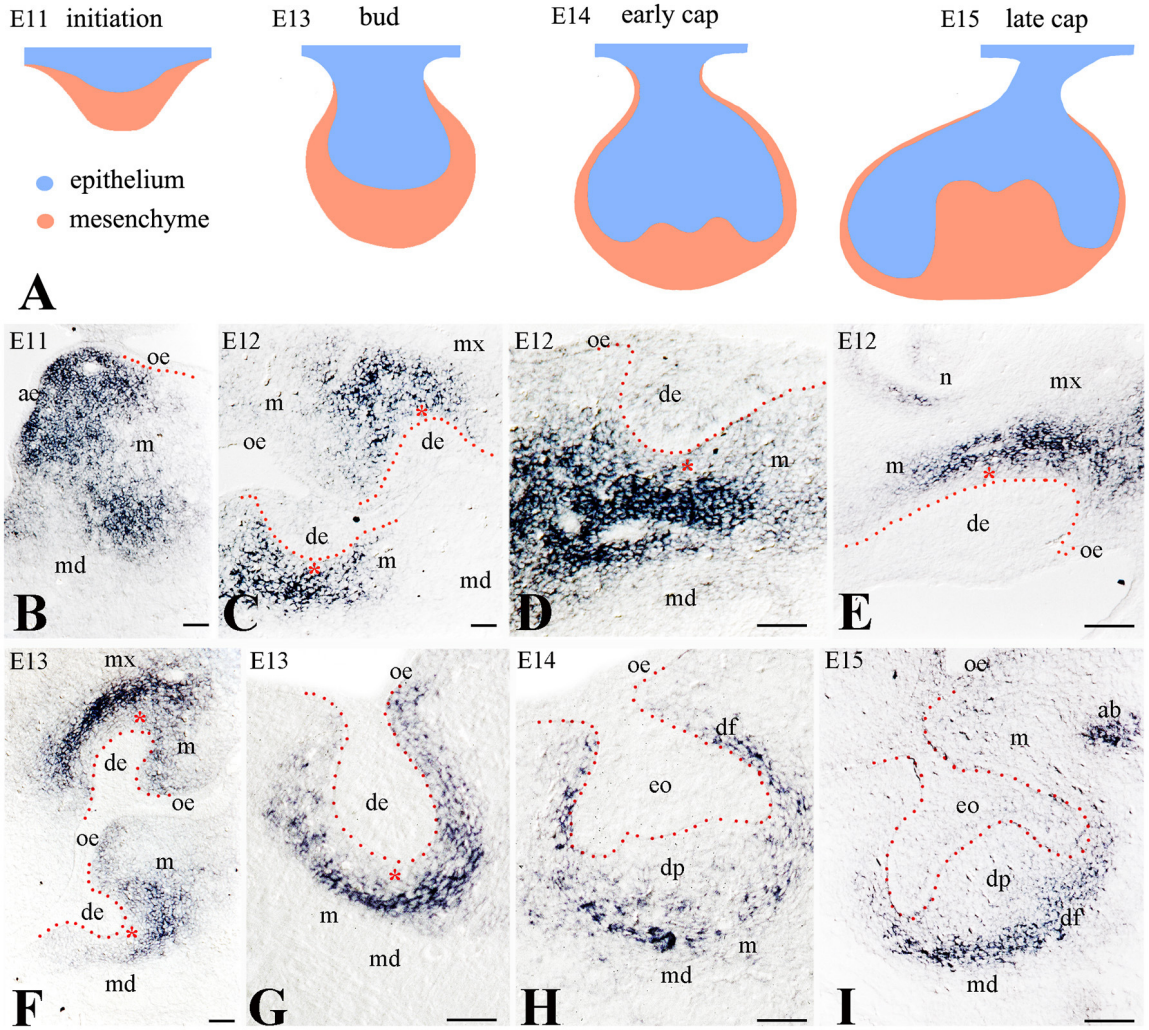
Expression of *Frzb* transcript in developing tooth germs by digoxigenin *in situ* hybridization on cryosections **(A)** Schematic representation of the early developmental tooth stages (E11–E15). **(B)** At E11, *Frzb* is expressed in the mandibular and maxillary mesenchyme, while it is excluded from the tooth germ. **(C–G)** At the bud stage (E12–E13), *Frzb* is expressed in the dental mesenchyme, but excluded from the 1–3 cell layers immediately adjacent to the dental epithelium (red asterisks), both in molars **(C,D,G)** and incisors **(E,F)**. **(H,I)** At the cap stage (E14–E15), *Frzb* expression consistently marks the dental follicle, while it is absent from the dental papilla. ab, alveolar bone; ae, aboral epithelium; de, dental epithelium; df, dental follicle; dp, dental papilla; eo, enamel organ; m, mesenchyme; md, mandible; mx, maxilla; oe, oral epithelium; n, nose. Scale bars: 100 μm.

To determine the potential role of Frzb in odontogenesis, we analyzed its expression pattern during the early stages of mouse tooth development (Figure 1A). We monitored the expression of *Frzb* in the developing mouse tooth germs from embryonic day 11 (E11; initiation stage) to E15 (cap stage). Intense hybridization signal was observed in the mesenchyme of the mandible during the tooth initiation period (E11) (Figure 1B). During the dental epithelial invagination to the underlying mesenchyme (early bud stage, E12), *Frzb* mRNA was restricted in mesenchymal cells located at the areas of molar (Figures 1C,D) and incisor (Figure 1E) formation. At this stage, the hybridization signal was strikingly absent from a layer of mesenchymal cells nearby the epithelium (Figures 1C–E, red asterisk). However, *Frzb* was strongly expressed in mesenchymal cells that are not in close contact with the dental epithelium (Figures 1C–E). This observation was confirmed by transcript localization at E13 (late bud stage) (Figures 1F,G). At the cap stage (E14–E15), *Frzb* hybridization signal was absent from the cells composing the dental papilla, while *Frzb* expression was strong in the peripheral regions of the developing tooth germ (Figures 1H,I).

### Differential proliferation of presumptive dental papilla and follicle cells

**Figure 2:**
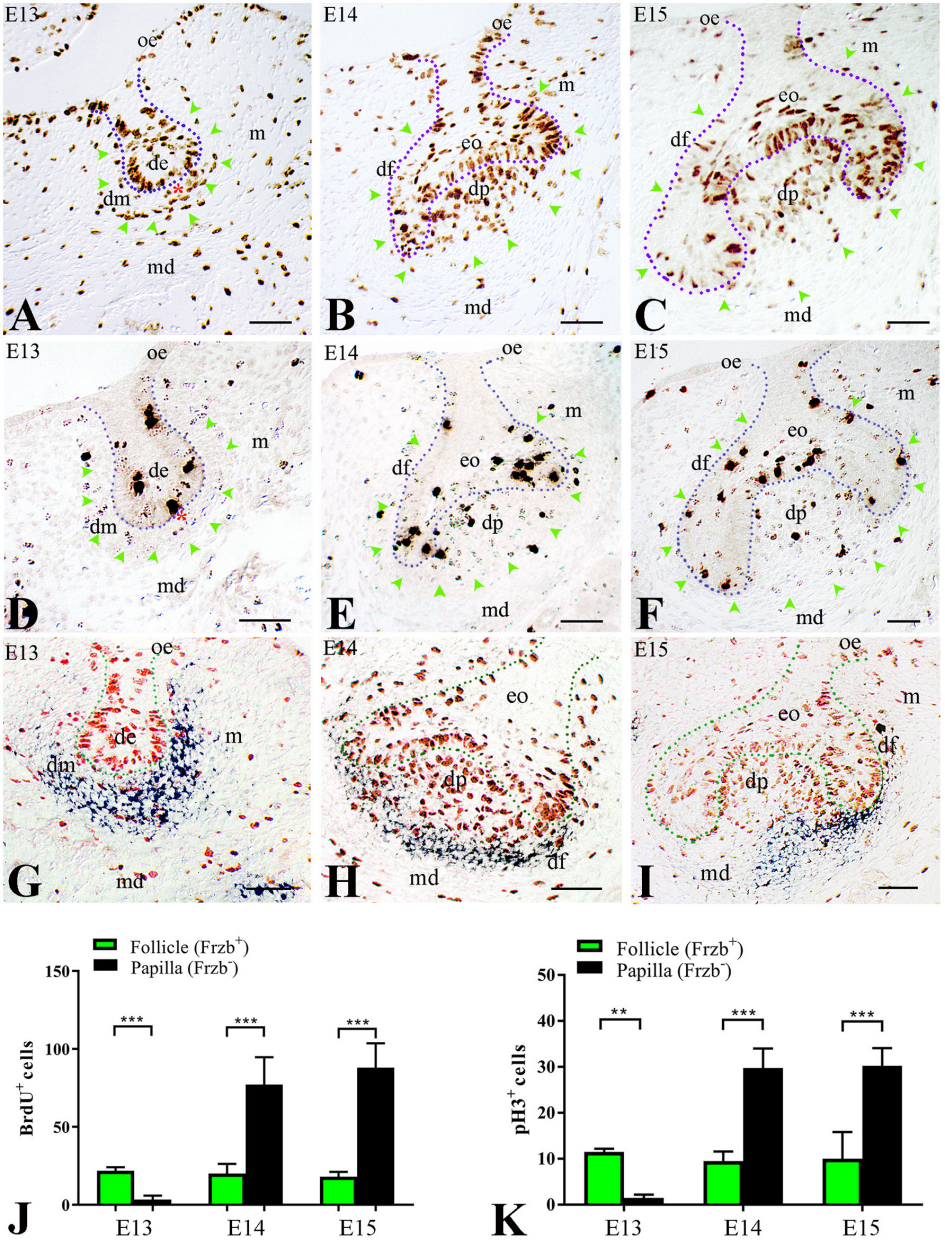
Differential proliferative behavior of dental mesenchymal populations. **(A)** BrdU immunostaining showing intense proliferation in the presumptive dental follicle region (green arrowheads), and little proliferation in the presumptive dental papilla (red asterisk). **(B,C)** At E14–E15, cell proliferation is concentrated in the dental papilla, while it is very limited in the dental follicle (green arrowheads). **(D)** At E13, phosphorylated Histone H3 (pH3) immunostaining indicates cell mitosis in the presumptive dental follicle region (green arrowheads), and little mitotic activity in the presumptive dental papilla (red asterisk). **(E,F)** At E14 and E15, pH3 immunolabelling shows intense mitotic activity in dental papilla and moderate in dental follicle. **(G–I)** Combined BrdU immunostaining (red) and *Frzb in situ* hybridization (blue) shows the correlation between cell proliferation and *Frzb* expression within the dental follicle and papilla at E13 **(G)**, E14 **(H)** and E15 **(I)**. **(J)** Quantification of BrdU^+^ cells in dental follicle and dental papilla at E13, E14, and E15. *n* = 5 vs. 5. **(K)** Quantification of pH3^+^ cells in the dental follicle and dental papilla at E13, E14, and E15 (*n* = 5 vs. 5). de, dental epithelium; df, dental follicle; dm, dental mesenchyme; dp, dental papilla; eo, enamel organ; m, mesenchyme; md, mandible; oe, oral epithelium. Scale bars: 100 μm. ^**^p < 0.001; ^***^p < 0.0001.

We then wondered whether this distinct *Frzb* expression pattern in the developing dental mesenchyme could correlate with dissimilar proliferative behavior between these two cell populations (*Frzb* expressing and *Frzb* non-expressing cells). To test this hypothesis, pregnant females were injected with BrdU and tooth germs of E13–E15 embryos were analyzed for cell proliferation. At E13 (bud stage), two territories could be observed according to BrdU immunoreactivity. Cell proliferation was significantly lower in the mesenchyme nearby the epithelium (Figure 2A, red asterisk), which does not express *Frzb* (Frzb^−^, Figure 2G), when compared to the mesenchyme that is most distant from the epithelium (Figure 2A; green arrowheads) and expresses *Frzb* (*Frzb*^+^, Figures 2G,J). At the subsequent cap stage (E14–E15), the proliferation status of the two cell populations switched: abundant mitotic activity was observed in mesenchymal cells forming the dental papilla (Figures 2B,C; green arrowheads) outside the Frzb expression domain (*Frzb*^−^, Figures 2H,I). In contrast, proliferative activity was sporadically detected in the forming dental follicle (Figures 2B,C; green arrowheads) that expresses *Frzb* (*Frzb*^+^, Figures 2H–J). To confirm the differential proliferation status between cells of the dental follicle (*Frzb*^+^ cells) and dental papilla (*Frzb*^−^ cells), we stained E13–E15 tooth germs for phospho-Histone H3 (pH3), which marks cells in active mitosis (M phase) (Figures 2D–F). pH3 immunohistochemistry showed that *Frzb*^+^ cells of the dental follicle proliferate significantly more than *Frzb*^−^ cells of the presumptive dental papilla at E13 (Figures 2D,K). A proliferative switch occurred at E14–E15, when *Frzb*^+^ cells of the follicle display a significantly lower mitotic activity than *Frzb*^−^ cells of the papilla (Figures 2F,I,K).

### *Frzb* expressing cells mark the presumptive dental follicle

**Figure 3:**
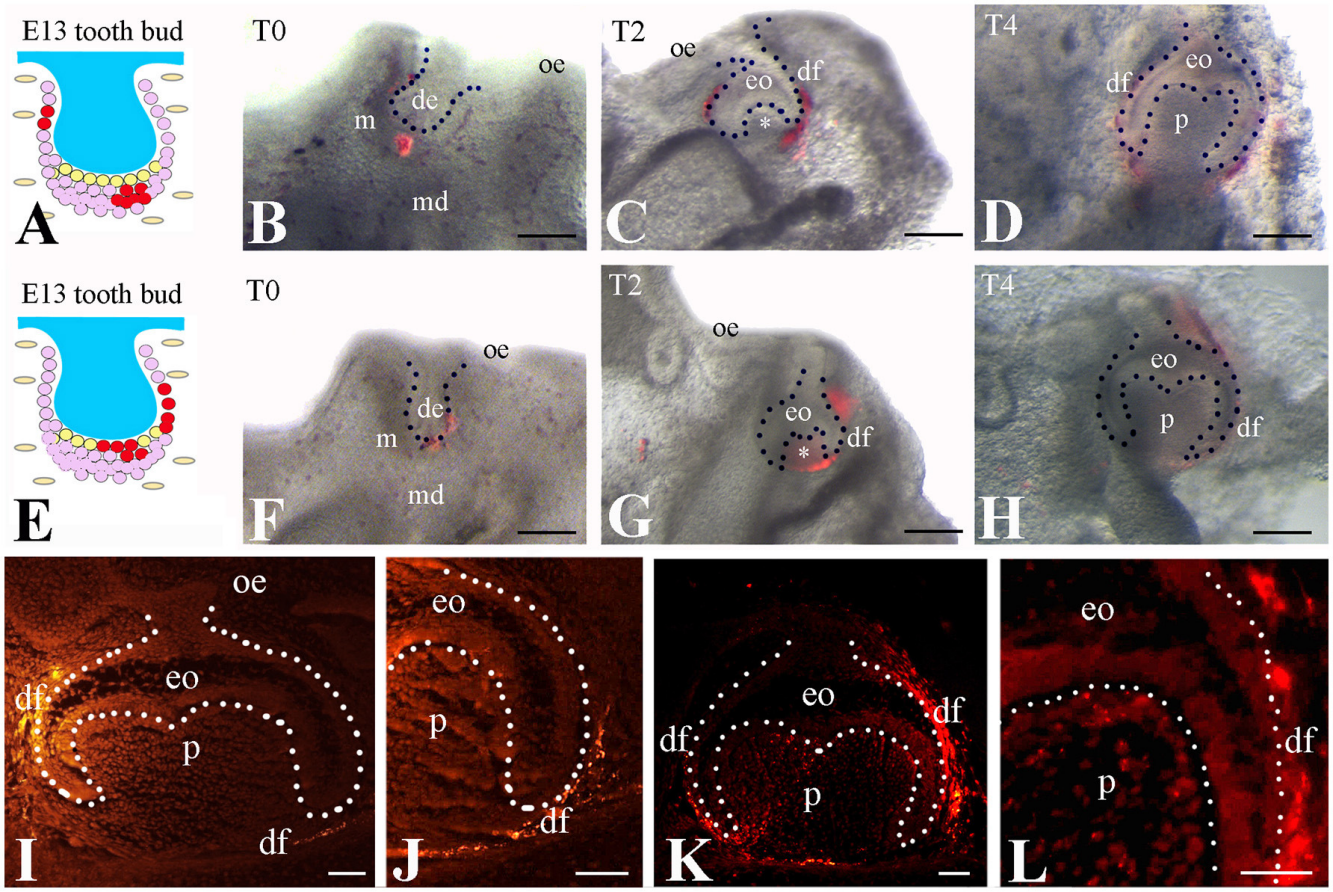
Tracing of DiI-labeled cells from the *Frzb*^+^ and the *Frzb*^−^ mesenchymal territories. **(A–D)** DiI**-**tracing of presumptive follicular cells (*n* > 10 per location of injection). DiI was injected in the dental mesenchymal region distant from the dental epithelium **(A,B)**. After 2 **(C)** and 4 **(D)** days, the marked cells and their progeny gave rise uniquely to the dental follicle, as no fluorescence could be detected within the dental papilla (white asterisk in **C**). **(E–H)** DiI-tracing of mesenchymal cells both distant and adjacent to the dental epithelium. DiI was injected in close proximity to the dental epithelium **(E,F)**. After 2 **(G)** and 4 **(H)** days, DiI labeling could be observed in the papilla (white asterisk in **(G)** and in the dental follicle domain. **(I–L)** Dark field pictures showing DiI-tracing of distant mesenchymal cells (see **A**) at day 4. When DiI was injected at a distance from the dental epithelium, DiI^+^-cells (bright yellow color) were observed only in the dental follicle **(I,J)**. Worth note that some epithelial cells next to the dental follicle were also injected with DiI during the procedure. In contrast, when both distant and adjacent mesenchymal cells were injected with DiI, fluorescence was observed in the dental pulp and dental follicle **(K,L)**. de, dental epithelium; df, dental follicle; eo, enamel organ; m, mesenchyme; md, mandible; oe, oral epithelium; p, pulp. Scale bars: **(B–D/F–H)** 200 μm; **(I–L)**: 100 μm.

To test if the *Frzb* expressing cells selectively contribute to the formation of dental follicle, we labeled subsets of the *Frzb* expressing (Figure 3A) and non-expressing (Figure 3E) mesenchymal domains with DiI at the bud stage and followed the fate of DiI-positive cells (Figures 3B–D,F–H). DiI-positive cells that were located in the territory of *Frzb* expression participated in the formation of the dental follicle of the cap (2 days of culture) and early bell (4 days of culture) staged teeth (Figures 3B–D). As DiI does not allow simultaneous labeling of the entire *Frzb* expression domain, we marked different sub-regions in a significant number of experiments (*n* > 10). In all experiments realized, DiI staining was never observed neither in the dental papilla compartment of these developing teeth, nor in other tissues formed far away from the tooth germ (Figures 3C,D,I,J). Conversely, DiI labeled cells in direct contact with the dental epithelium (*Frzb*^−^ cells) contributed to dental papilla formation at the cap (2 days of culture) and early bell (4 days of culture) stages (*n* > 10; Figures 3F–H). Sections of the tooth germs of the early bell stage indicated dental papilla cells stained with DiI (Figures 3G,H,K,L). Since DiI injection was performed in both tooth mesenchymal cell populations in this experiment, DiI labeled cells were present in both dental follicle and dental papilla cells. Due to the intrinsic limitations of this technique we rarely obtained DiI labeling in dental epithelial cells when injection was performed in mesenchymal cells immediately adjacent to dental epithelial bud (Figures 3E,F,I).

## Discussion

**Figure 4:**
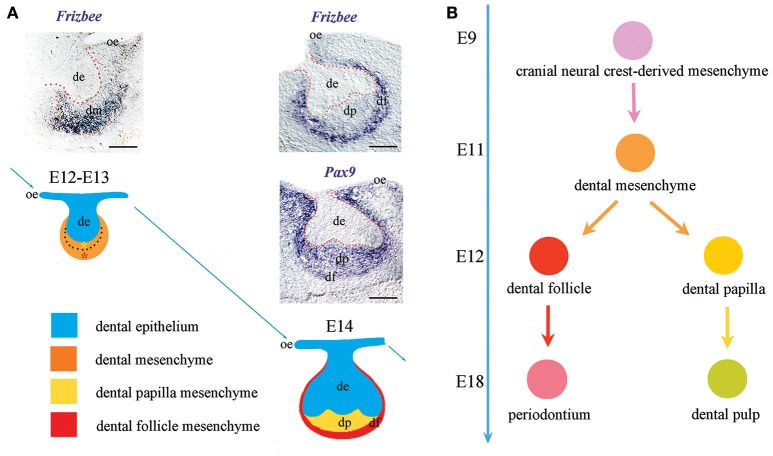
*Frzb* expression determinates the presumptive periodontal territory from the earliest stages of odontogenesis. **(A)** Differently from well-established dental mesenchymal markers (e.g., *Pax9*), *Frzb* is expressed solely in the territory that will give rise to the periodontium from the very beginning of odontogenesis. **(B)** *Frzb* expression pattern suggests that the commitment of dental mesenchymal cells toward either follicle/periodontium or papilla/pulp formation occurs already from the bud stage of tooth development (E12–E13). Scale bars: 200 μm.

Cranial neural crest-derived mesenchymal cells play a crucial role in tooth formation and are recognizable since the earliest stages of odontogenesis due to their positivity to classical neural crest or dental mesenchyme-specific markers such as *Pax9* (Bonczek et al., 2017), *Barx1* (Mitsiadis et al., 1998), and *midkine (MK)* (Mitsiadis et al., 1995). These cells have been historically considered as a homogeneous population of cells; nevertheless, the dental mesenchyme generates highly specialized adult soft tissues, such as the pulp and the periodontium (Jiménez-Rojo et al., 2014; Otsu et al., 2014; Mitsiadis et al., 2015). It is therefore of great interest the discovery of new markers that would allow distinguishing the subsets of mesenchymal cells possessing different differentiation commitments. Here we identify *Frzb* as a novel dental mesenchymal marker. Importantly, *Frzb* is specifically expressed from the earliest tooth developmental stages in mesenchymal cells that are not in direct contact with the dental epithelium. This specific expression pattern only partially overlaps with the expression domain of other well-established dental mesenchymal markers, such as *Pax9* (Figure 4A). One previous report described the expression of *Frzb* in the dental mesenchyme (Sarkar and Sharpe, 1999). However, the radioactive *in situ* hybridization technique that was used for the detection of the transcripts did not offer sufficient resolution to distinguish the absence of *Frzb* mRNA expression in the cell layers immediately adjacent to the dental epithelium. Based on *Frzb* expression we could identify two functionally distinct cell domains: one expressing *Frzb* that will give rise to the dental follicle, and another non-expressing *Frzb* that will form the dental papilla. This important finding shows for the first time the presence of two functionally distinct cell populations in the tooth mesenchyme and their commitment for generating specialized dental structures of the tooth at very early developmental stages (Figure 4B). During early odontogenesis (E11–E12), the thickened dental epithelium is surrounded by mesenchymal cells that condense and actively proliferate (Mitsiadis et al., 2003; Mitsiadis and Graf, 2009). These combined activities of cell migration, adhesion and proliferation within the mesenchyme are controlled by signaling and cell adhesion molecules such as fibroblast growth factors, midkine, syndecan. Dental mesenchyme is important for the tooth shape determination that involves alternated and well-orchestrated proliferations of distinct cell populations throughout odontogenesis. Initially, active proliferative events in the territory that will give rise to dental follicle/periodontium correlate with *Frzb* expression. This initial process of active cell proliferation within the *Frzb* expression domain determines the size of the periodontal domain that is important for proper tooth germ development and its integration in the growing surrounding environment (e.g., alveolar bone). Subsequently, once *Frzb* expressing cells have delimited the dental papilla domain, cells from this tiny *Frzb*-negative territory start to actively proliferate in order to increase the tissue size. This process is accompanied by morphological rearrangements within the growing epithelium that dictate the tooth shape.

The differential proliferative activities between dental pulp cells and dental follicle cells are not only limited to development. In our previous studies, we have shown that when pulp and follicle cells are co-cultured, they never intermingle and they assume an organization reminiscent of the *in vivo* situation, with follicle cells surrounding and engulfing pulp cells (Schiraldi et al., 2012). These cell populations appear to have a clear genetic memory and compete for their own territory even *in vitro*. Our results indicate that these two cell populations are functionally and molecularly discriminated from the earliest stages of odontogenesis, thus providing a developmental basis to their clear and persistent peculiarity. Future studies are required to understand the molecular mechanisms underlying the early specification and fate of these cell populations. In this regard, *Frzb*-based genetic lineage tracing fluorescent analysis on transgenic mouse models might be instrumental to understand the exact contribution of *Frzb*-expressing cells to tooth development as well as to the formation and the regeneration of the periodontium. Since Frzb is an extracellular Wnt antagonist, it is tempting to speculate that the restricted expression of *Frzb* to a subset of mesenchymal cells could generate an asymmetric barrier whose function is to limit the activation of the Wnt cascade only in certain cells. It is plausible, in fact, that distinct differentiation fates require cells to be Wnt-responsive, while others necessitate the inhibition of this pathway, as it occurs for the early development of the mouse heart (Marvin et al., 2001) and the eye lens (Cantù et al., 2014; Cvekl and Ashery-Padan, 2014). In this scenario, Frzb might restrict the activity of the secreted Wnt ligands to the oral epithelium and to the presumptive dental papilla. Consistently, many genes encoding for Wnt ligands are specifically expressed from the oral epithelium throughout tooth development (Sarkar and Sharpe, 1999) and genetic and molecular evidence indicates that the epithelium must remain responsive to canonical Wnt signaling. When a mutated, transcriptionally silent, version of β-catenin is exclusively expressed in the epithelium (i.e., epithelial cells become unresponsive to canonical Wnt signaling), tooth development stops abruptly (Cantù et al., 2017). Intriguingly, *Frzb* expression coincides with the dental mesenchymal territory that receives the growing neurons (Pagella et al., 2014). Indeed, from the earliest stages of odontogenesis, nerve fibers grow toward the tooth germ and progressively innervate the dental follicle. On the contrary, these nerves do not penetrate the dental papilla mesenchyme until the late stage of tooth mineralization (Pagella et al., 2014). It is thus possible that *Frzb* expression could regulate tooth innervation and probably morphogenesis. Indeed, increasing evidence indicates that innervation plays an active role in organ morphogenesis and development (Kumar and Brockes, 2012; Pagella et al., 2014). Taken together the present results suggest that the developing dental follicle necessitates a specific inhibition of the activity of the Wnt ligands. Conditional knockout and overexpression studies will constitute fundamental approaches to determine the exact role of Frzb in odontogenesis.

Both the dental pulp (formed by the *Frzb* non-expressing dental papilla cells) and the periodontal ligament (formed by the *Frzb* expressing dental follicle) contain stem cells, the dental pulp and the periodontal ligament stem cell populations, which hold the promise of regenerative approaches (Huang et al., 2009; Mitsiadis et al., 2011; Jiménez-Rojo et al., 2014). The present findings increase the arsenal of markers that permits the specific isolation of mesenchymal cells with desired differentiation potential by the combined use of mesenchymal markers. An increased knowledge of the regulation of mesenchymal dental cells will allow the future development of novel treatments for dental tissue repair and regeneration.

## Author contributions

TM designed the project, performed the experiments, interpreted the data, and wrote the manuscript; PP and CC contributed to the interpretation of the data and wrote the manuscript.

### Conflict of interest statement

The authors declare that the research was conducted in the absence of any commercial or financial relationships that could be construed as a potential conflict of interest.

## References

[B1] Aurrekoetxea M. Irastorza I. García-Gallastegui P. Jiménez-Rojo L. Nakamura T. Yamada Y.. (2016). Wnt/β-catenin regulates the activity of epiprofin/Sp6, SHH, FGF, and BMP to coordinate the stages of odontogenesis. Front. Cell Dev. Biol. 4:25. 10.3389/fcell.2016.00025 27066482 PMC4811915

[B2] Aurrekoetxea M. Lopez J. García P. Ibarretxe G. Unda F. (2012). Enhanced Wnt/β-catenin signalling during tooth morphogenesis impedes cell differentiation and leads to alterations in the structure and mineralisation of the adult tooth. Biol. Cell 104, 603–617. 10.1111/boc.201100075 22671936

[B3] Babb R. Chandrasekaran D. Carvalho Moreno Neves V. Sharpe P. T. (2017). Axin2-expressing cells differentiate into reparative odontoblasts via autocrine Wnt/β-catenin signaling in response to tooth damage. Sci. Rep. 7:3102. 10.1038/s41598-017-03145-6 28596530 PMC5465208

[B4] Balic A. Thesleff I. (2015). Tissue interactions regulating tooth development and renewal. Curr. Top. Dev. Biol. 115, 157–186. 10.1016/bs.ctdb.2015.07.006 26589925

[B5] Bonczek O. Balcar V. J. Sery O. (2017). PAX9 gene mutations and tooth agenesis: a review. Clin. Genet. 92, 467–476. 10.1111/cge.12986 28155232

[B6] Cantù C. Pagella P. Shajiei T. D. Zimmerli D. Valenta T. Hausmann G.. (2017). A cytoplasmic role of Wnt/β-catenin transcriptional cofactors Bcl9, Bcl9l, and Pygopus in tooth enamel formation. Sci. Signal. 10, 1–11. 10.1126/scisignal.aah4598 28174279

[B7] Cantù C. Zimmerli D. Hausmann G. Valenta T. Moor A. Aguet M.. (2014). Pax6-dependent, but β-catenin-independent, function of Bcl9 proteins in mouse lens development. Genes Dev. 28, 1879–1884. 10.1101/gad.246140.114 25184676 PMC4197948

[B8] Clevers H. (2006). Wnt/β-catenin signaling in development and disease. Cell 127, 469–480. 10.1016/j.cell.2006.10.018 17081971

[B9] Cruciat C. M. Niehrs C. (2013). Secreted and transmembrane wnt inhibitors and activators. Cold Spring Harb. Perspect. Biol. 5, 1–26. 10.1101/cshperspect.a015081 23085770 PMC3578365

[B10] Cvekl A. Ashery-Padan R. (2014). The cellular and molecular mechanisms of vertebrate lens development. Development 141, 4432–4447. 10.1242/dev.107953 25406393 PMC4302924

[B11] Gruenbaum-Cohen Y. Tucker A. S. Haze A. Shilo D. Taylor A. L. Shay B. (2009). Amelogenin in cranio-facial development: the tooth as a model to study the role of amelogenin during embryogenesis. J. Exp. Zool. B Mol. Dev. Evol. 312, 445–457. 10.1002/jez.b.21255 19097165

[B12] Hoang B. Moos M. Vukicevic S. Luyten F. P. (1996). Primary structure and tissue distribution of *frzb*, a novel protein related to Drosophila frizzled, suggest a role in skeletal morphogenesis. J. Biol. Chem. 271, 26131–26137. 10.1074/jbc.271.42.26131 8824257

[B13] Huang G. T.-J. Gronthos S. Shi S. (2009). Mesenchymal stem cells derived from dental tissues vs. those from other sources: their biology and role in regenerative medicine. J. Dent. Res. 88, 792–806. 10.1177/0022034509340867 19767575 PMC2830488

[B14] Jiménez-Rojo L. Granchi Z. Woloszyk A. Filatova A. Pagella P. Mitsiadis T. A. (2014). Regenerative dentistry: stem cells meet nanotechnology, in Horizons in Clinical Nanomedicine, 1st Edn., eds Karagkiozaki V. Logothetidis S. (Singapore: Pan Standford Publishing), 255–288.

[B15] Jussila M. Thesleff I. (2012). Signaling networks regulating tooth organogenesis and regeneration, and the specification of dental mesenchymal and epithelial cell lineages. Cold Spring Harb. Perspect. Biol. 4:a008425. 10.1101/cshperspect.a008425 22415375 PMC3312678

[B16] Krivanek J. Adameyko I. Fried K. (2017). Heterogeneity and developmental connections between cell types inhabiting teeth. Front. Physiol. 8:386. 10.3389/fphys.2017.00376 28638345 PMC5461273

[B17] Kumar A. Brockes J. P. (2012). Nerve dependence in tissue, organ, and appendage regeneration. Trends Neurosci. 35, 691–699. 10.1016/j.tins.2012.08.003 22989534

[B18] Leyns L. Bouwmeester T. Kim S. H. Piccolo S. De Robertis E. M. (1997). Frzb-1 is a secreted antagonist of Wnt signaling expressed in the Spemann organizer. Cell 88, 747–756. 10.1016/S0092-8674(00)81921-2 9118218 PMC3061830

[B19] Liu F. Chu E. Y. Watt B. Zhang Y. Gallant N. M. Andl T.. (2008). Wnt/beta-catenin signaling directs multiple stages of tooth morphogenesis. Dev. Biol. 313, 210–224. 10.1016/j.ydbio.2007.10.016 18022614 PMC2843623

[B20] Liu W. Konermann A. Guo T. Jäger A. Zhang L. Jin Y. (2014). Canonical Wnt signaling differently modulates osteogenic differentiation of mesenchymal stem cells derived from bone marrow and from periodontal ligament under in fl ammatory conditions. Biochim. Biophys. Acta 1840, 1125–1134. 10.1016/j.bbagen.2013.11.003 24231680

[B21] Marvin M. J. Di Rocco G. Gardiner A. Bush S. M. Lassar A. B. (2001). Inhibition of Wnt activity induces heart formation from posterior mesoderm. Genes Dev. 15, 316–327. 10.1101/gad.855501 11159912 PMC312622

[B22] Mitsiadis T. A. Graf D. (2009). Cell fate determination during tooth development and regeneration. Birth Defects Res. C Embryo Today 87, 199–211. 10.1002/bdrc.20160 19750524

[B23] Mitsiadis T. A. Luder H. U. (2011). Genetic basis for tooth malformations: from mice to men and back again. Clin. Genet. 80, 319–329. 10.1111/j.1399-0004.2011.01762.x 21819395

[B24] Mitsiadis T. A. Chéraud Y. Sharpe P. Fontaine-Pérus J. (2003). Development of teeth in chick embryos after mouse neural crest transplantations. Proc. Natl. Acad. Sci. U.S.A. 100, 6541–6545. 10.1073/pnas.1137104100 12740432 PMC164482

[B25] Mitsiadis T. A. Feki A. Papaccio G. Catón J. (2011). Dental pulp stem cells, niches, and notch signaling in tooth injury. Adv. Dent. Res. 23, 275–279. 10.1177/0022034511405386 21677078

[B26] Mitsiadis T. A. Mucchielli M. L. Raffo S. Proust J. P. Koopman P. Goridis C. (1998). Expression of the transcription factors Otlx2, Barx1 and Sox9 during mouse odontogenesis. Eur. J. Oral Sci. 106, 112–116. 10.1111/j.1600-0722.1998.tb02161.x 9541211

[B27] Mitsiadis T. A. Orsini G. Jimenez-Rojo L. (2015). Stem cell-based approaches in dentistry. Eur. Cells Mater. 30, 248–257. 10.22203/eCM.v030a17 26562631

[B28] Mitsiadis T. A. Salmivirta M. Muramatsu T. Muramatsu H. Rauvala H. Lehtonen E.. (1995). Expression of the heparin-binding cytokines, midkine (MK) and HB-GAM (pleiotrophin) is associated with epithelial-mesenchymal interactions during fetal development and organogenesis. Development 121, 37–51. 7867507 10.1242/dev.121.1.37

[B29] Mitsiadis T. A. Tucker A. S. De Bari C. Cobourne M. T. Rice D. P. C. (2008). A regulatory relationship between Tbx1 and FGF signaling during tooth morphogenesis and ameloblast lineage determination. Dev. Biol. 320, 39–48. 10.1016/j.ydbio.2008.04.006 18572158

[B30] Otsu K. Kumakami-Sakano M. Fujiwara N. Kikuchi K. Keller L. Lesot H.. (2014). Stem cell sources for tooth regeneration: current status and future prospects. Front. Physiol. 5:36. 10.3389/fphys.2014.00036 24550845 PMC3912331

[B31] Pagella P. Jiménez-Rojo L. Mitsiadis T. A. (2014). Roles of innervation in developing and regenerating orofacial tissues. Cell. Mol. Life Sci. 71, 2241–2251. 10.1007/s00018-013-1549-0 24395053 PMC11113802

[B32] Person A. D. Garriock R. J. Krieg P. A. Runyan R. B. Klewer S. E. (2005). Frzb modulates Wnt-9a-mediated β-catenin signaling during avian atrioventricular cardiac cushion development. Dev. Biol. 278, 35–48. 10.1016/j.ydbio.2004.10.013 15649459

[B33] Sarkar L. Sharpe P. T. (1999). Expression of Wnt signalling pathway genes during tooth development. Mech. Dev. 85, 197–200. 10.1016/S0925-4773(99)00095-7 10415363

[B34] Schiraldi C. Stellavato A. D'Agostino A. Tirino V. D'Aquino R. Woloszyk A.. (2012). Fighting for territories: time-lapse analysis of dental pulp and dental follicle stem cells in co-culture reveals specific migratory capabilities. Eur. Cell. Mater. 24, 426–440. 10.22203/eCM.v024a30 23180452

[B35] Tamura M. Nemoto E. (2016). Role of the Wnt signaling molecules in the tooth. Jpn. Dent. Sci. Rev. 52, 75–83. 10.1016/j.jdsr.2016.04.001 28408959 PMC5390339

[B36] Zhang L. Liu W. Zhao J. Ma X. Shen L. Zhang Y.. (2016). Mechanical stress regulates osteogenic differentiation and RANKL / OPG ratio in periodontal ligament stem cells by the Wnt/β-catenin pathway. Biochim. Biophys. Acta 1860, 2211–2219. 10.1016/j.bbagen.2016.05.003 27154288

[B37] Zimmerli D. Hausmann G. Cantù C. Basler K. (2017). Pharmacological interventions in the Wnt pathway: inhibition of Wnt secretion versus disrupting the protein-protein interfaces of nuclear factors. Br. J. Pharmacol. [Epub ahead of print]. 10.1111/bph.13864 28521071 PMC5727313

